# What do Cochrane systematic reviews say about interventions for insomnia?

**DOI:** 10.1590/1516-3180.2018.0380311018

**Published:** 2018-12-13

**Authors:** Florence de Lucca Melo, Juan Fulgencio Welko Mendoza, Carolina de Oliveira Cruz Latorraca, Rafael Leite Pacheco, Ana Luiza Cabrera Martimbianco, Daniela Vianna Pachito, Rachel Riera

**Affiliations:** I Undergraduate Medical Student, Escola Paulista de Medicina (EPM), Universidade Federal de São Paulo (UNIFESP), São Paulo (SP), Brazil.; II Undergraduate Medical Student, Escola Paulista de Medicina (EPM), Universidade Federal de São Paulo (UNIFESP), São Paulo (SP), Brazil.; III MSc. Psychologist; Postgraduate Student, Evidence-Based Health Program, Universidade Federal de São Paulo (UNIFESP); and Assistant Researcher, Cochrane Brazil, São Paulo (SP), Brazil.; IV MD. Postgraduate Student, Evidence-Based Health Program, Universidade Federal de São Paulo (UNIFESP), and Assistant Researcher, Cochrane Brazil, São Paulo (SP), Brazil.; V MSc, PhD. Physiotherapist; Postdoctoral Student, Evidence-Based Health Program, Universidade Federal de São Paulo (UNIFESP); Professor, Health and Environment Program, Universidade Metropolitana de Santos (UNIMES); and Volunteer Researcher, Cochrane Brazil, São Paulo (SP), Brazil.; VI MD, MSc. Neurologist; Postgraduate Student, Evidence-Based Health Program, Universidade Federal de São Paulo (UNIFESP); and Assistant Researcher, Cochrane Brazil, São Paulo (SP), Brazil.; VII MD, MSc, PhD. Rheumatologist; Adjunct Professor, Discipline of Evidence-Based Medicine, Escola Paulista de Medicina (EPM), Universidade Federal de São Paulo (UNIFESP); and Researcher, Cochrane Brazil, São Paulo (SP), Brazil.

**Keywords:** Review [publication type]. Evidence-based medicine. Evidence-based practice. Sleep Initiation and Maintenance Disorders. Comparative Effectiveness Research

## Abstract

**CONTEXT AND OBJECTIVE:**

Insomnia is a frequent complaint that generates more than five million visits to doctors per year in the United States. This study summarizes all Cochrane systematic reviews (SRs) that evaluated interventions to treat insomnia.

**DESIGN AND SETTING:**

Review of SRs, conducted in the Discipline of Evidence-Based Medicine, Escola Paulista de Medicina (EPM), Universidade Federal de São Paulo (UNIFESP).

**METHODS:**

A sensitive search was carried out in the Cochrane Database of Systematic Reviews to identify Cochrane SRs that assessed the effects of any type of intervention for people with insomnia. The results, main characteristics of the SRs and the certainty of the evidence obtained from them were synthesized and discussed.

**RESULTS:**

Seven SRs were included. They addressed the benefits and harm of acupuncture (n = 1), behavioral interventions (n = 1), music (n = 1), pharmacotherapy (n = 2), phototherapy (n = 1) and physical exercise (n = 1). The certainty of the evidence ranged from moderate to very low.

**CONCLUSION:**

Acupuncture, music, physical exercise, paroxetine, doxepin, trimipramine and trazodone seem to present some benefit for patients with insomnia. However, the uncertainty around these results means that no robust and definitive recommendations for clinical practice can be made until the benefits and harms from each intervention for patients with insomnia have been confirmed through further studies.

## INTRODUCTION

Insomnia is a frequent complaint that generates more than five million visits to doctors per year in the United States.^1^ It is considered to be a subjective condition that affects sleep maintenance, onset or early waking.^2^ It is also a public health issue because of its impact on people’s wellbeing.^3^

Patients with insomnia usually present difficulty in initiating or maintaining sleep and may wake up without the capacity to go back to sleep. This situation may induce development of symptoms during the day, such as sleepiness, mood disturbances and fatigue.^4^^,^^5^

Insomnia may precede or appear along with other diagnoses and may occur as a result of different stressors. The following individual factors are commonly associated with the risk of insomnia: female sex, older age (for any sex), previous episodes of insomnia and family history.^6^^,^^7^

It is classified as a disorder and may be divided into primary or secondary according to the Diagnostic and Statistical Manual of Mental Disorders, Fifth Edition (DSM-5). Primary insomnia is more common and secondary insomnia is associated with another disorder.^4^^,^^5^ Acute or short-term insomnia is considered to consist of at least three nights of difficulty in sleeping for no more than three months. If no treatment is provided, it may progress to chronic insomnia, which is a stage of the disorder that is more difficult to treat.^8^

Insomnia may be a risk for developing hypertension, diabetes, obesity and cardiovascular diseases.^9^^,^^10^^,^^11^^,^^12^ It may also increase the risk of psychiatric comorbidities and substance abuse and it reduces the quality of life.^13^^,^^14^^,^^15^

The treatments available include pharmacological options, psychological interventions and alternative therapies, but all of these may have limitations. Physicians need to take into account the symptoms relating to the insomnia, treatment availability, effectiveness and safety.

## OBJECTIVE

The aim of this study was to make a synthesis of the evidence from Cochrane systematic reviews that assessed different therapies for insomnia and to discuss this evidence.

## METHODS

### Design

Review of Cochrane systematic reviews (SRs).

### Setting

Discipline of Evidence-Based Medicine, Escola Paulista de Medicina (EPM), Universidade Federal de São Paulo (UNIFESP).

### Criteria for including reviews

#### Types of studies

We considered the latest version of Cochrane SRs. We did not include any protocols or any systematic reviews (SRs) that had been withdrawn from the Cochrane Database of Systematic Reviews (CDSR).

#### Types of participants

We considered participants with insomnia. We excluded SRs in which was not possible to identify insomnia as the type of sleep disturbance presented by the participant. We did not impose any restriction based on age or sex.

#### Types of intervention

We considered any surgical, pharmacological or non-pharmacological intervention.

#### Types of outcomes

We considered any outcomes that had been evaluated by the authors of the SRs included. These outcomes included any clinical, social, laboratory or economic outcomes that had been reported.

### Search for reviews

We conducted a broad and unrestricted systematic search in the Cochrane Database of Systematic Reviews (via Wiley) on August 1, 2018. The search strategy is presented in Table 1.

**Table 1.** Search strategy

#1 MeSH descriptor: [Sleep Initiation and Maintenance Disorders] explode all trees
#2 (Disorders of Initiating and Maintaining Sleep) OR (DIMS (Disorders of Initiating and Maintaining Sleep)) OR (Early Awakening) OR (Awakening, Early) OR (Nonorganic Insomnia) OR (Insomnia, Nonorganic) OR (Primary Insomnia) OR (Insomnia, Primary) OR (Transient Insomnia) OR (Insomnia, Transient) OR (Rebound Insomnia) OR (Insomnia, Rebound) OR (Secondary Insomnia) OR (Insomnia, Secondary) OR (Sleep Initiation Dysfunction) OR (Dysfunction, Sleep Initiation) OR (Dysfunctions, Sleep Initiation) OR (Sleep Initiation Dysfunctions) OR (Sleeplessness) OR (Insomnia Disorder) OR (Insomnia Disorders) OR (Insomnia) OR (Insomnias) OR (Chronic Insomnia) OR (Insomnia, Chronic) OR (Psychophysiological Insomnia) OR (Insomnia, Psychophysiological)
#3 #1 OR #2
#4 #3 in Cochrane Reviews

### Selection of systematic reviews

The selection phase consisted of independent reading of all the abstracts retrieved, by two researchers, to check their eligibility in relation to the inclusion criteria. Any disagreement was resolved through reaching a consensus or by consulting a third author.

### Presentation of the results

We made a synthesis of all the SRs included and presented the key results and methodological issues using a narrative approach (qualitative synthesis).

The SRs included were summarized based on the following characteristics:

- Inclusion criteria/PICO (population, intervention, comparator and outcomes)
- Methodological issues relating to searching for and coding of studies
- Main results
- Critical assessment of studies included (risk-of-bias assessment)
- Analyses performed (including methods for pooling research through meta-analysis)
- Assessment of the certainty of the evidence using the GRADE approach.^16^
- Applicability

When the SRs included considered multiple interventions, we presented only those that were relevant for this review.

## RESULTS

### Search results

The search strategy retrieved 80 systematic reviews (SRs). After the screening and selection process, seven SRs were included and a synthesis was produced from them.^17^^,^^18^^,^^19^^,^^20^^,^^21^^,^^22^^,^^23^

### Results from systematic reviews

The SRs included assessed acupuncture (n = 1), behavioral interventions (n = 1), music (n = 1), pharmacotherapy (n = 2), phototherapy (n = 1) and physical exercise (n = 1). The main findings and the certainty of the evidence (according to GRADE)^16^ for all the SRs included are shown in Table 2.

**Table 2.** Characteristics of interventions for insomnia: comparisons, outcomes and certainty of evidence

Intervention	Comparisons	Population	Main findings	GRADE
Acupuncture^17^	Acupuncture with or without association with other interventions versus placebo or sham or no treatment	Adults	Acupressure versus no treatment/sham/placebo: benefit from acupuncture regarding sleep quality Acupuncture as adjunct to other treatment versus other treatment alone: benefit from acupuncture regarding sleep quality	Not assessed
Antidepressants^18^	Serotonin reuptake inhibitors versus placebo	Adults	Paroxetine: benefit regarding sleep quality	Very low
			Fluoxetine: no important difference between groups	Low
	Tricyclic antidepressants versus placebo		Doxepin or trimipramine: benefit regarding subjective sleep quality, sleep efficiency and duration of sleep no difference regarding sleep latency or adverse events	
				Moderate
				Moderate/low
	“Other” antidepressants versus placebo		Trazodone: Benefit regarding subjective sleep outcomes No benefit regarding sleep efficiency Worse on adverse events	
				Moderate
				Low
				Low
Bright light therapy^19^	Bright light therapy versus any other active intervention, no intervention or sham	Adults aged 60 or above	No study found	---
Cognitive behavioral intervention^20^	Cognitive behavioral intervention versus no intervention, use of placebo or patient on waiting list	Adults aged 60 or above	No difference in sleep onset latency or duration of sleep	Not assessed
Melatonin^22^	Melatonin (2 mg of slow-released melatonin, once daily, before bedtime) versus placebo	Adults with dementia due to Alzheimer’s disease	No difference in caregivers’ sleep quality or in activities of daily living	Not assessed**
Music^21^	Listening to pre-recorded music versus no intervention	Adults	Benefit regarding subjective sleep quality	Moderate
			No difference in sleep onset latency, total duration of sleep, sleep interruption or sleep efficiency	Low
Physical exercise^23^	16 weeks of moderate-intensity community-based exercise training versus no treatment (patient on waiting list)	Adults aged 60 or above	Benefit regarding sleep quality	Not assessed
Trazodone^22^	Trazodone (50 mg) versus placebo	Adults with dementia due to Alzheimer’s disease	Benefit regarding total nocturnal sleep time and sleep efficiency	Low
			No difference in time spent awake after sleep onset, number of nocturnal awakenings, daytime sleep or activities of daily living	Low

*GRADE (Grading of Recommendations Assessment, Development and Evaluation) has the aim of assessing the certainty of the evidence. From this, the results are classified as having high certainty of evidence (high confidence that the estimated effect is close to the true effect); moderate certainty of evidence (likely that the estimated effect is close to the real effect, but there is a possibility that it is not); low certainty of evidence (limited confidence in the effect estimate) or very low certainty of evidence (the true effect is likely to be substantially different from the estimate effect). **The certainty of the evidence was graded considering all studies on melatonin that were included in the review. However, only one of them was on insomnia (the other studies considered all types of sleep disturbance. Thus, the GRADE that was specific for this study was not assessed.

### Acupuncture

The aim of this review^17^ was to determine the efficacy and safety of acupuncture for insomnia. The authors compared groups receiving the same interventions with or without associated acupuncture, against placebo or sham or no treatment. Randomized controlled trials (RCTs) that compared different acupuncture methods or acupuncture against other treatments were not considered. The review included 33 RCTs (2293 participants, aged 15 to 98 years) that had assessed needle acupuncture, electroacupuncture, acupressure or magnetic acupressure. The main results are presented below.

#### Acupressure versus no treatment/sham/placebo

Acupressure resulted in a benefit regarding sleep quality, compared with no treatment (odds ratio, OR = 13.08; 95% confidence interval, CI = 1.79 to 95.59; 2 RCTs; 280 participants) or sham/placebo (OR = 6.62; 95% CI = 1.78 to 24.55; 2 RCTs; 112 participants). However, in a sensitivity analysis in which an assumption was made that dropouts had worse outcomes, acupuncture ceased to be conclusively beneficial.

#### Acupuncture as an adjunct to another treatment versus this other treatment alone

Acupuncture as an adjunct to another treatment was found to present the possibility of benefit regarding sleep quality (OR = 3.08; 95% CI = 1.93 to 4.90; 13 RCTs; 883 participants,). In subgroup analyses, needle acupuncture alone was shown to be beneficial, but not electroacupuncture.

All the trials presented high risk of bias and were heterogeneous regarding the definition of insomnia, participant characteristics, acupoints and scheme of treatment. Overall, the effect sizes were small, with wide CIs. A risk of publication bias was detected and adverse events were poorly reported and were rare. For further details, refer to the original abstract, available at: https://www.cochranelibrary.com/cdsr/doi/10.1002/14651858.CD005472.pub3/full.

### Antidepressants for treating insomnia in adults

The aim of this review^18^ was to assess the effects of antidepressants for treating insomnia. Twenty-three RCTs were included (2806 participants), comparing any antidepressant with placebo, other medications for insomnia or a different antidepressant.

#### Serotonin reuptake inhibitors (SSRIs) versus placebo

- Paroxetine: significant improvements on the Pittsburgh Sleep Quality Index scale at six weeks (2 RCTs; 60 participants; p = 0.03) and 12 weeks (2 RCTs; 27 participants; p < 0.001).
- Fluoxetine: no important difference between groups.

There was also no proper reporting of adverse events. The overall certainty of evidence for the subjective outcomes ranged from low to very low.

#### Tricyclic antidepressants (TCA) versus placebo

Six RCTs (812 participants) compared tricyclic antidepressants (TCA) with placebo (five used doxepin and one used trimipramine). No trials for amitriptyline were found. The main results are presented below.

- Subjective sleep quality: benefit from TCA (standardized mean difference, SMD = -0.39; 95% CI = -0.56 to -0.21; 5 RCTs; 518 participants).
- Sleep efficiency: benefit from TCA (mean difference, MD = 6.29 percentage points; 95% CI = 3.17 to 9.41; 4 RCTs; 510 participants).
- Duration of sleep: benefit from TCA (MD = 22.88 minutes; 95% CI = 13.17 to 32.59; 4 RCTs; 510 participants).
- Sleep latency (time taken to fall asleep): no difference between groups (MD = 0.27 minutes; 95% CI = -9.01 to 0.48; 4 RCTs; 510 participants).
- Adverse events: no difference between groups (risk ratio, RR = 1.02; 95% CI = 0.86 to 1.21; 6 RCTs; 812 participants).

#### Other antidepressants versus placebo

Eight RCTs compared other antidepressants with placebo (one used mianserin and seven used trazodone). The main results are presented below.

- Subjective sleep outcomes: benefit from trazodone (SMD = -0.34; 95% CI = -0.66 to -0.02; 3 RCTs; 370 participants).
- Sleep efficiency (measured using polysomnography): no difference between trazodone and placebo (MD = 1.38 percentage points; 95% CI = -2.87 to 5.63; 2 RCTs; 169 participants).
- Adverse effects: more frequent with trazodone than with placebo (no numerical data provided; 2 RCTs).

The authors concluded that the effects of SSRIs were uncertain, compared with placebo. There may have been be a small improvement in sleep quality with short-term use of low-dose doxepin and trazodone. There was no evidence of any effect in relation to use of amitriptyline or long-term antidepressants for treating insomnia. The tolerability and safety of antidepressants for treating insomnia were also uncertain. For further details, refer to the original abstract, available at: http://cochranelibrary-wiley.com/doi/10.1002/14651858.CD010753.pub2/full#CD010753-sec1-0017.

### Bright light therapy for sleep problems in adults aged 60+

“Bright light therapy” (“phototherapy”) involves administration of high‐intensity light (frequently, 10,000 lux at the point of impact) for set periods of time with the aim of synchronizing sleep onset to socially acceptable norms. This review^19^ aimed to evaluate the effects of bright light therapy on improving sleep quality among adults aged 60 and above. The reviewers did not find any RCTs on which to base conclusions regarding the effectiveness of this treatment. Future RCTs to evaluate this intervention are imperative and, until then, bright light therapy cannot be indicated routinely for treating sleep problems among adults aged 60 years and over. For further details, refer to the original abstract, available at: http://cochranelibrary-wiley.com/doi/10.1002/14651858.CD003403/full.

### Cognitive behavioral interventions

Cognitive and behavioral approaches towards sleep problems include making changes to poor sleep habits, promoting better sleep hygiene practices and challenging negative thoughts, beliefs and behaviors relating to sleep. These therapeutic options, if beneficial and safe, could have advantages over pharmacological approaches, which are frequently associated with undesirable events.

The purpose of this review^20^ was to assess the efficacy and safety of cognitive-behavioral interventions (CBT) among older people (older than 60 years) with insomnia. All forms of CBT were considered, including sleep hygiene, stimulus control, muscle relaxation, sleep restriction and cognitive therapies alone. CBT was compared with no intervention, waiting-list control and/or placebo (“quasi-desensitization”). Six randomized clinical trials (RCTs) were included (282 participants). The main results are presented below.

- Sleep onset latency (time taken to fall asleep), as reported in participants’ diaries: no difference between CBT and control groups immediately post-intervention (mean difference, MD, in time to sleep onset = -3 minutes; 95% confidence interval, CI = -8.92 to 2.92; 3 RCTs; 135 participants; certainty of evidence not assessed) or at 12 months or more after treatment (MD = -11.48 minutes; 95% CI = -23.54 to 0.58; 1 RCT; 74 participants; certainty of evidence not assessed).
- Sleep onset latency (time taken to fall asleep), as measured by polysomnography: no difference between CBT and control groups immediately post-intervention (MD = 4.3 minutes; 95% CI = -13.29 to 4.55; 1 RCT; 24 participants; certainty of evidence not assessed) or at 12 months or more after treatment (MD = 2.49 minutes; 95% CI = -3.24 to 8.22; 1 RCT; 74 participants; certainty of evidence not assessed).
- Sleep duration (total, in minutes), as reported in participants’ diaries: no difference between CBT and control groups immediately post-intervention (MD in time to sleep onset = -14.56 minutes; 95% CI = -36.13 to 7.01; 4 RCTs; 153 participants; certainty of evidence not assessed), at 3 months (MD = 14.77 minutes; 95% CI = -38.96 to 68.50; 1 RCT; 26 participants; certainty of evidence not assessed) or at 12 months or more after treatment (MD = -31.49 minutes; 95% CI = -71.11 to 8.13; 1 RCT; 50 participants; certainty of evidence not assessed).

The benefits associated with CBT regarding waking after sleep onset were probably clinically modest. The data were based on a single study with a small-sized sample regarding total time awake. For further details, refer to the original abstract, available at: http://cochranelibrary-wiley.com/doi/10.1002/14651858.CD003161/full.

### Music for insomnia among adults

This review^21^ assessed the effects of listening to music on insomnia among adults. Six RCTs and quasi-randomized controlled trials (qRCTs) were included, comprising 314 participants. The studies compared the effects of listening to music daily at one’s own house with no treatment or treatment-as-usual, on sleep improvement among adults with insomnia. The main results are presented below.

- Sleep quality as assessed using the Pittsburgh Sleep Quality Index (scale from 0 to 21 scale, on which lower scores mean better sleep quality): benefit from listening to music (MD = -2.80; 95% CI = -3.42 to -2.17; 5 studies; 264 participants; moderate certainty of evidence).
- Sleep onset latency, total duration of sleep, sleep interruption and sleep efficiency assessed using a questionnaire in the morning, with evaluations on participants using polysomnography. One study (50 participants) reported results from these outcomes but did not provide sufficient numerical data for analysis. The authors of that study only reported that there was no evidence of any effect from the intervention. The certainty of evidence regarding these outcomes was downgraded due to risk of bias and imprecision.
- Adverse events: none of the studies included reported this outcome.

The authors of this review^21^ concluded that music may be effective for improving subjective sleep quality among adults with insomnia. They also highlighted that this intervention is easy to administer. It is imperative that further RCTs should be conducted to increase the certainty of the evidence and to investigate more aspects of this intervention. For further details, refer to the original abstract, available at: http://cochranelibrary-wiley.com/doi/10.1002/14651858.CD010459.pub2/full.

### Pharmacotherapies for sleep disturbances in dementia

This review^22^ evaluated the effects of any drug treatment versus placebo for sleep disorders among people with dementia. Six RCTs were eligible for inclusion, but only two included participants with insomnia as a sleep disturbance, and these assessed melatonin (15 participants; 1 RCT) and trazodone (30 participants; 1 RCT). All the participants had dementia due to Alzheimer’s disease (AD), in association with their insomnia. The main results are presented below.

#### Melatonin (2 mg of slow-released melatonin, once daily, before bedtime) versus placebo

- Caregivers’ sleep quality (change from baseline): no difference between groups (MD = 0.74 minutes; 95% CI = - 0.40 to 1.89; 2 RCTs; 15 participants).
- Activities of daily living (change from baseline): no difference between groups (MD = - 0.66; 95% CI = -1.9 to 0.58; 15 participants; 1 RCT).

No serious adverse effects from melatonin were reported in the studies included.

#### Trazodone (50 mg) versus placebo (for two weeks)

- Total nocturnal duration of sleep: benefit of trazodone (MD = 42.46 minutes; 95% CI = 0.9 to 84.0; 30 participants; 1 RCT).
- Sleep efficiency: benefit from trazodone (MD = 8.53%; 95% CI = 1.9 to 15.1; 30 participants; 1 RCT).
- Time spent awake after sleep onset: no difference between groups (MD = -20.41; 95% CI = -60.4 to 19.6; 30 participants; 1 RCT).
- Number of nocturnal awakenings: no difference between groups (MD = -3.71; 95% CI = -8.2 to 0.8; 30 participants; 1 RCT).

No effects on daytime sleep, cognition or activities of daily living were found. No serious adverse effects from trazodone were reported.

For further details, refer to the original abstract, available at: http://cochranelibrary-wiley.com/doi/10.1002/14651858.CD009178.pub3/full.

### Physical exercise for treating sleep problems among adults aged 60+

This review^23^ assessed physical exercise among older adults (60 years and over). Only one RCT was included (43 participants). The patients received 16 weeks of moderate-intensity community-based exercise training or no treatment (these patients were on a waiting list).

Compared with the control group, the patients in the exercise training group showed significant improvement in the Pittsburgh Sleep Quality Index (scale from 0 to 21, on which lower scores mean better sleep quality) at 16 weeks (MD = -3.4 points; 95% CI = -1.9 to -5.4; one RCT; 43 participants).

The reports on the other sleep-related outcomes were not presented numerically. Moreover, this review was published in 2002 and therefore needs to be updated. It also did not assess any safety outcomes and this should be considered in further studies. For further details, refer to the original abstract, available at: http://cochranelibrary-wiley.com/doi/10.1002/14651858.CD003404/full.

## DISCUSSION

The present review included seven Cochrane systematic reviews (SRs) that assessed the effects of different interventions for insomnia in the general population or among groups with specific disorders (people with dementia and the elderly). Overall, the current evidence from Cochrane SRs shows that acupuncture, music, physical exercise, paroxetine, doxepin, trimipramine and trazodone seem to present some benefit for patients with insomnia. However, the certainty of the evidence provided by these SRs ranged from very low to moderate, which means that it is likely or very likely that further studies may change the current evidence.

Three of the SRs included are out of date, since they were published in 2002 and 2003.^19^^,^^20^^,^^23^ Updates for these reviews are urgently needed, to aid in searching for new studies and to revise the analyses in line with newer recommendations.

There are seven ongoing Cochrane SRs (protocols) that will be published in the future. Their aims are to assess new non‐benzodiazepine hypnotics (eszopiclone, zaleplon, zolpidem and zopiclone),^24^^,^^25^^,^^26^^,^^27^ ramelteon (melatonin receptor agonist)^28^ and pharmacological and non-pharmacological interventions for treating insomnia during pregnancy.^29^ Additionally, a network meta-analysis is being conducting to compare the efficacy and acceptability of all pharmacological treatments for insomnia among adults.^30^ These SRs will be useful both for healthcare professionals and for patients, to aid in decision-making.

## CONCLUSION

This review identified seven Cochrane systematic reviews that assessed pharmacological or non-pharmacological interventions for treating insomnia. Based on their findings, acupuncture, music, physical exercise, paroxetine, doxepin, trimipramine and trazodone seem to present some benefit for patients with insomnia. However, the uncertainty relating to these results means that no robust and definitive recommendations for clinical practice can be made until the benefits and harm from of each intervention for patients with insomnia have been confirmed through further studies.
